# Efficacy of aspergillomarasmine A/meropenem combinations with and without avibactam against bacterial strains producing multiple β-lactamases

**DOI:** 10.1128/aac.00272-24

**Published:** 2024-08-12

**Authors:** Caitlyn M. Rotondo, Gerard D. Wright

**Affiliations:** 1 David Braley Centre for Antibiotic Discovery, McMaster University, Hamilton, Ontario, Canada; 2 M.G. DeGroote Institute for Infectious Disease Research, McMaster University, Hamilton, Ontario, Canada; 3 Department of Biochemistry and Biomedical Sciences, McMaster University, Hamilton, Ontario, Canada; University of Fribourg, Fribourg, Switzerland

**Keywords:** antibiotic resistance, aspergillomarasmine A, beta-lactams, carbapenems, cephems, penams, metallo-beta-lactamases, serine-beta-lactamases

## Abstract

The effectiveness of β-lactam antibiotics is increasingly threatened by resistant bacteria that harbor hydrolytic β-lactamase enzymes. Depending on the class of β-lactamase present, β-lactam hydrolysis can occur through one of two general molecular mechanisms. Metallo-β-lactamases (MBLs) require active site Zn^2+^ ions, whereas serine-β-lactamases (SBLs) deploy a catalytic serine residue. The result in both cases is drug inactivation via the opening of the β-lactam warhead of the antibiotic. MBLs confer resistance to most β-lactams and are non-susceptible to SBL inhibitors, including recently approved diazabicyclooctanes, such as avibactam; consequently, these enzymes represent a growing threat to public health. Aspergillomarasmine A (AMA), a fungal natural product, can rescue the activity of the β-lactam antibiotic meropenem against MBL-expressing bacterial strains. However, the effectiveness of this β-lactam/β-lactamase inhibitor combination against bacteria producing multiple β-lactamases remains unknown. We systematically investigated the efficacy of AMA/meropenem combination therapy with and without avibactam against 10 *Escherichia coli* and 10 *Klebsiella pneumoniae* laboratory strains tandemly expressing single MBL and SBL enzymes. Cell-based assays demonstrated that laboratory strains producing NDM-1 and KPC-2 carbapenemases were resistant to the AMA/meropenem combination but became drug-susceptible upon adding avibactam. We also probed these combinations against 30 clinical isolates expressing multiple β-lactamases. *E. coli*, *Enterobacter cloacae,* and *K. pneumoniae* clinical isolates were more susceptible to AMA, avibactam, and meropenem than *Pseudomonas aeruginosa* and *Acinetobacter baumannii* isolates. Overall, the results demonstrate that a triple combination of AMA/avibactam/meropenem has potential for empirical treatment of infections caused by multiple β-lactamase-producing bacteria, especially Enterobacterales.

## INTRODUCTION

The use of antibiotics selects for resistance mechanisms that readily spread among bacterial pathogens (1, 2). Consequently, many bacterial isolates now demonstrate resistance to multiple antibiotics (3, 4). These multidrug-resistant (MDR) pathogens threaten the effectiveness of antibiotics, such as β-lactams, resulting in one of the biggest threats to public health in the 21^st^ century (5). There is an urgent need to preserve the activity of existing antibiotics to circumvent the threat posed by MDR bacteria.

The predominant resistance mechanism to β-lactams is enzyme-catalyzed inactivation by β-lactamases. These enzymes are divided into four classes based on their sequence identity and mechanism of action. β-Lactamases utilize either an active site serine residue (class A, C, and D serine-β-lactamases) or active site Zn^2+^ ions (class B metallo-β-lactamases) to inactivate β-lactams by hydrolyzing the β-lactam warhead essential to their activity (1, 6). An increasing number of bacteria contain multiple β-lactamases from different classes (7, 8). Therefore, combination therapy, which uses two or more antibiotics and/or resistance inhibitors to target these β-lactamase-producing bacteria, is increasingly common in recovering the activity of β-lactam antibiotics. A significant advantage of this approach is synergy, where the combined effect of two or more antibiotics and/or inhibitors is greater than the sum of their individual activities (4, 9). Given the rise of MDR strains and the growing diversity of β-lactamases, the discovery of a therapy that targets bacteria producing any combination of all four classes of β-lactamases would be the most advantageous.

Several serine-β-lactamase (SBL) inhibitors have been developed for clinical use (e.g., avibactam, vaborbactam, clavulanic acid) in fixed-dose combinations with various β-lactam antibiotics (10, 11). However, metallo-β-lactamases (MBLs) remain a clinical challenge due to their insensitivity to approved SBL inhibitors and their ability to hydrolyze most β-lactam antibiotics (12, 13). Aspergillomarasmine A (AMA) is a natural product synthesized by *Aspergillus versicolor* (14). Although AMA exhibits no antimicrobial activity, its inhibitory potency increases when combined with β-lactam antibiotics, especially carbapenems, such as meropenem (15). Indeed, AMA was shown to restore the activity of meropenem against Enterobacteriaceae, *Acinetobacter*, and *Pseudomonas* isolates, producing either NDM-1 or VIM-2, two clinically relevant MBLs (14).

Although AMA offers a means to mitigate the presence of MBLs, given the growing number of MDR isolates expressing both MBLs and SBLs, adding an SBL inhibitor provides a strategy to expand the effectiveness of an AMA/meropenem combination therapy. This study used isogenic *Escherichia coli* and *Klebsiella pneumoniae* laboratory strains expressing individual MBL and SBL genes together with MDR clinical isolates to evaluate the AMA/meropenem pairing efficacy. The inhibitory potency of different AMA/β-lactam combinations was also explored in the presence of avibactam, which can inhibit several SBL enzymes (16). The resulting data provide a road map to study the effect of combining MBL and SBL inhibitors in treating infections caused by bacteria producing multiple β-lactamases.

## RESULTS

### Construction of tandem β-lactamase gene expression plasmids

We previously determined that AMA can rescue meropenem activity in MBL-producing bacteria (14, 15). However, the efficiency of an AMA/meropenem combination against bacterial strains producing multiple β-lactamases remains unknown. To investigate this activity, one MBL (NDM-1) and one SBL (KPC-2) were cloned into the low-copy number plasmid pGDP2 in tandem, creating NDM-1/KPC-2 and KPC-2/NDM-1 ordered constructs (17). These plasmids contained a P_Lac_ promoter upstream of both genes (Fig. S1). Once transformed into *E. coli* BW25113, we determined the minimum inhibitory concentration (MIC) for different β-lactam antibiotics to investigate gene expression.

Under individual promoter control, the NDM-1/KPC-2 and KPC-2/NDM-1 constructs had identical meropenem, piperacillin, cefepime, and cefaclor MIC values and similar doripenem and cefotaxime MIC values (Table S1). These results are consistent with the observation that these two β-lactamase enzymes confer resistance to these antibiotics (Table S2). To determine if the genes were being expressed equally in the constructs, we evaluated the MIC for aztreonam, which is known to be susceptible to KPC-2 but not NDM-1 (Table S2). The NDM-1/KPC-2 and KPC-2/NDM-1 constructs demonstrated respective aztreonam MIC values of 128 and 256 µg/mL, indicating similar KPC expression (Table S1).

### Inhibition of multiple β-lactamases by the AMA/meropenem combination is dependent on β-lactamase class and carbapenemase activity

We previously demonstrated that the MBL inhibitory potency of AMA varied with the β-lactam partner, and that the optimal antibiotic was a carbapenem, such as meropenem (15). To follow up on this finding, we sought to determine the efficacy of an AMA/meropenem combination against 10 *E. coli* BW25113 and 10 *K*. *pneumoniae* ATCC 33495 strains, producing one MBL and one SBL. These included common β-lactamases from each class, such as KPC-2 and CTX-M-15 (class A), NDM-1 (class B), CMY-2 (class C), and OXA-48 (class D) (18–22). In addition, certain strains were designed to produce OXA-23, which is among the most frequently isolated carbapenem-hydrolyzing class D β-lactamase and a predominant cause of carbapenem resistance in *A. baumannii* isolates worldwide (23).

Most *E. coli* and *K. pneumoniae* strains required an AMA concentration of 8–16 µg/mL to restore the activity of meropenem to its susceptibility breakpoint (Tables 1 and 2). The NDM-1/KPC-2 and KPC-2/NDM-1 constructs demonstrated the highest resistance level, requiring a concentration of AMA of >32 µg/mL to restore susceptibility to meropenem (Tables 1 and 2), consistent with the carbapenemase activity of KPC-2 (Table S2). Furthermore, OXA-23 and OXA-48 showed little to no activity against meropenem and doripenem when produced by *E. coli* BW25113 (Table S2) and had only modest resistance in *K. pneumoniae* (Table S3). Singkham-in et al. have reported that carbapenem susceptibility varied between different *K. pneumoniae* isolates in a strain-specific manner partially related to gene expression levels (24). Overall, the results demonstrated that strains expressing SBL carbapenemases (e.g., KPC-2) are resistant to the AMA/meropenem combination and may also require the presence of an SBL inhibitor (e.g., avibactam) to alleviate β-lactam resistance.

**TABLE 1:** Concentration of AMA needed to restore the activity of different β-lactam antibiotics to their susceptibility breakpoint concentration against *E. coli* BW25113 strains producing one MBL and one SBL*^a^*^,^*^i^*^,^*^j^*

β-Lactamase ^*b*^		[AMA] at the susceptibility breakpoint of the antibiotics in different combinations (µg/mL)^*c*^^,*d*^								
Gene 1	Gene 2	AMA/ MEM	AVI/ MEM	AMA/AVI/ MEM	AMA/AVI/ DOR	AMA/AVI/ PIP	AMA/AVI/ AMP	AMA/AVI/ CTX	AMA/AVI/ FEP	AMA/AVI/ CEC
NDM-1^*f*^	KPC-2^*e*^	> 32	16	8	8	16	> 32	32	16	> 32
NDM-1	CTX-M-15^*e*^	16	16	8	8	16	> 32	32	16	> 32
NDM-1	CMY-2^*g*^	16	16	8	8	16	> 32	32	16	> 32
NDM-1	OXA-23^*h*^	16	16	16	16	32	> 32	32	16	> 32
NDM-1	OXA-48^*h*^	16	16	8	16	16	> 32	32	16	> 32

^*a*^ Both β-lactamase genes were cloned into pGDP2 with individual promoters.

^*b*^ Because similar results were obtained regardless of the position of the β-lactamase genes, the data for the constructs with NDM-1 at position two were removed from the table.

^*c*^ The EUCAST susceptibility breakpoint concentrations for MEM, DOR, PIP, AMP, CTX, FEP, and CEC are 2, 1, 8, 8, 1, 1, and 1 µg/mL.

^*d*^ AVI was maintained at 4 µg/mL, except during the AVI/MEM combination.

^*e*^ Class A.

^*f*^ Class B.

^*g*^ Class C.

^*h*^ Class D.

^*i*^ AMA, aspergillomarasmine A; AVI, avibactam; MEM, meropenem; DOR, doripenem; PIP, piperacillin; AMP, ampicillin; CTX, cefotaxime; FEP, cefepime; CEC, cefaclor.

^*j*^ All assays were conducted in duplicate.

**TABLE 2:** Concentration of AMA needed to restore the activity of meropenem to its susceptibility breakpoint concentration against *K. pneumoniae* ATCC 33495 strains producing one MBL and one SBL*^a^*^,^*^i^*^,^*^j^*

β-Lactamase ^*b*^		[AMA] at 2 µg/mL of MEM in different combinations (µg/mL)^*c*^^,*d*^		
Gene 1	Gene 2	AMA/ MEM	AVI/ MEM	AMA/AVI/ MEM
NDM-1^*f*^	KPC-2^*e*^	> 64	> 64	8
NDM-1	CTX-M-15^*e*^	16	> 64	16
NDM-1	CMY-2^*g*^	8	> 64	8
NDM-1	OXA-23^*h*^	16	> 64	16
NDM-1	OXA-48^*h*^	8	> 64	16

^*a*^ Both β-lactamase genes were cloned into pGDP2 with individual promoters.

^*b*^ Because similar results were obtained regardless of the position of the β-lactamase genes, the data for the constructs with NDM-1 at position two were removed from the table.

^*c*^ 2 µg/mL is the EUCAST susceptibility breakpoint concentration for MEM.

^*d*^ AVI was maintained at 4 µg/mL, except during the AVI/MEM combination.

^*e*^ Class A.

^*f*^ Class B.

^*g*^ Class C.

^*h*^ Class D.

^*i*^ AMA, aspergillomarasmine A; AVI, avibactam; MEM, meropenem.

^*j*^ All assays were conducted in duplicate.

### β-Lactamase inhibitors showed a greater inhibitory potency when combined with a carbapenem antibiotic

To determine the optimal AMA/avibactam/β-lactam combination, we partnered AMA and avibactam with two carbapenems (meropenem, doripenem), two penams (piperacillin, ampicillin), and three cephems (cefotaxime, cefepime, cefaclor). The inhibitory potency of these combinations was evaluated against 10 laboratory *E. coli* strains, producing one MBL and one SBL. The results demonstrated that most *E. coli* strains required 8 µg/mL of AMA and 4 µg/mL of avibactam to restore susceptibility to both meropenem and doripenem (Table 1). For strains producing either NDM-1/KPC-2 or KPC-2/NDM-1, this is an improvement over the AMA/meropenem combination, where resistance to meropenem was observed even at 32 µg/mL of AMA. Furthermore, most strains generally become resensitized to piperacillin and cefepime at 16 µg/mL of AMA and 4 µg/mL of avibactam (Table 1). However, >32 µg/mL of AMA and 4 µg/mL of avibactam were necessary to restore susceptibility to ampicillin and cefaclor (Table 1). The wild-type *E. coli* BW25113 strain encodes a chromosomal cephalosporinase (AmpC). Therefore, these results are consistent with carbapenems, piperacillin, cefepime, and cefotaxime being poor substrates for AmpC, whereas ampicillin and older cephalosporins are susceptible to hydrolysis by this β-lactamase (25). The results indicated that AMA and avibactam achieved the greatest inhibitory potency when combined with a carbapenem antibiotic such as meropenem.

### The efficacy of the AMA/avibactam/meropenem combination depends on the class of β-lactamase and the bacterial order

The meropenem concentration chosen to evaluate the effectiveness of AMA and avibactam was 2 µg/mL, the European Committee on Antimicrobial Susceptibility Testing (EUCAST) susceptibility breakpoint of meropenem (26). These AMA/avibactam/meropenem potentiation assays were then evaluated against 10 *K*. *pneumoniae* ATCC 33495 strains producing one MBL and one SBL. Similar to the results against *E. coli*, all *K. pneumoniae* strains were resensitized to meropenem at 8–16 µg/mL of AMA and 4 µg/mL of avibactam (Table 2). However, more variability in the effectiveness of the AMA/avibactam/meropenem combination was observed when tested against 30 MDR clinical strains.

Several clinical strains became susceptible to meropenem at 8–16 µg/mL of AMA and 4 µg/mL of avibactam (Table 3). The results were similar whether these strains produced seven different β-lactamases (CTX-M-15, TEM-166, OXA-2, NDM-1, CMY-6, AmpC, and OXA-1), such as *E. coli* GN610 or just three β-lactamases (ACT-17, VIM-1, and AmpH), such as *E. cloacae* 397260. If no β-lactamases were being expressed, the AMA/avibactam/meropenem combination was not expected to affect the antibiotic susceptibility of these clinical strains. In addition, the results demonstrated that the addition of avibactam to the AMA/meropenem combination generally increased inhibitory potency against strains producing multiple class D OXA β-lactamases but had minimal effect on strains producing β-lactamases solely from classes A–C. Furthermore, AMA/avibactam/meropenem was less effective against *P. aeruginosa* isolates, requiring concentrations >64 µg/mL of AMA and 4 µg/mL of avibactam to restore meropenem susceptibility, even if only VIM-2 was being produced (e.g., *P. aeruginosa* H1010812). In addition, *A. baumannii* B1NG08a showed a high level of resistance to the AMA/ avibactam/meropenem combination (Table 3; Table S6) due to the production of OXA-23, a carbapenem-hydrolyzing class D β-lactamase, which is non-susceptible to AMA and avibactam (14, 27). Additional findings demonstrated that the efficacy of the AMA/avibactam/ meropenem combination was not necessarily related to the initial degree of avibactam resistance for the strains. For example, *E. coli* GN688 and *P. rettgeri* GN570 conferred avibactam MIC values of 16 and 1,024 µg/mL. Yet, both strains were resensitized to meropenem with 16 µg/mL of AMA and 4 µg/mL of avibactam (Table 3; Table S4). Overall, the results indicated that the AMA/avibactam/meropenem combination was effective against Enterobacterales producing β-lactamases from classes A–D but lacked activity against *Pseudomonas* and *Acinetobacter* isolates.

**TABLE 3:** Concentration of AMA needed to restore the activity of meropenem to its susceptibility breakpoint concentration in combination with and without avibactam against clinical strains producing multiple β-lactamases^*h*^^,*i*^

Strain^*a*^	β-Lactamases			[AMA] at 2 µg/mL of MEM (µg/mL)^*b*,*c*^		
				AMA/MEM	AVI/MEM	AMA/AVI/MEM
*Acinetobacter baumannii* B1NG08a	NDM-1^*e*^	OXA-23^*g*^	OXA-69^*g*^	> 64	> 64	> 64
*Citrobacter freundii* GN978	TEM-1^*d*^ NDM-1^*e*^ OXA-1^*g*^	CTX-M-15^*d*^ CMY-6^*f*^	CMY-46^*f*^	8	> 64	8
			CMY-18^*f*^			
*Enterobacter cloacae* 36749	NDM-1^*e*^	TEM-171^*d*^	OXA-1^*g*^	8	16	8
	AmpH^*f*^	ACT-25				
*Enterobacter cloacae* 47219	ACT-17^*f*^	NDM-1^*e*^	OXA-1^*g*^	8	32	16
	LAP-2^*d*^	TEM-1^*d*^	AmpH^*f*^			
*Enterobacter cloacae* 86502	ACT-17^*f*^	TEM-1^*d*^	CTX-M-15^*d*^	8	32	16
	OXA-1^*g*^	VIM-1^*e*^	AmpH^*f*^			
*Enterobacter cloacae* 397260	ACT-17^*f*^	VIM-1^*e*^	AmpH^*f*^	8	32	8
*Enterobacter cloacae* GN574	TEM-1^*d*^ OXA-1^*g*^	NDM-1^*e*^	CTX-M-15^*d*^	16	> 64	16
		ACT-17^*f*^				
*Enterobacter cloacae* GN579	CTX-M-15 ACT-25^*f*^	TEM-1 OXA-1	NDM-1^*e*^	16	32	8
*Enterobacter cloacae* GN687	NDM-1^*e*^ TEM-1^*d*^	OXA-9^*g*^ ACT-25^*f*^	CTX-M-15^*d*^	16	> 64	16
*Escherichia coli* 130392–1	TEM-1^*d*^ CMY-83^*f*^	NDM-5^*e*^	CMY-42^*f*^	32	32	32
*Escherichia coli* 376762	NDM-5^*e*^ TEM-1^*d*^ AmpC1^*f*^	CTX-M-15^*d*^ AmpH^*f*^	OXA-1^*g*^ AmpC^*f*^	32	8	32
*Escherichia coli* 376948	AmpC1^*f*^ TEM-1^*d*^	AmpH^*f*^ AmpC^*f*^	NDM-5^*e*^	32	8	> 64
*Escherichia coli* 387039	AmpH^*f*^ AmpC^*f*^ OXA-1^*g*^	NDM-5^*e*^ AmpC1^*f*^	OXA-181^*g*^ CMY-2^*f*^	> 64	64	64
*Escherichia coli* GN610	CTX-M-15^*d*^ NDM-1^*e*^ OXA-1^*g*^	TEM-166^*d*^ CMY-6^*f*^	OXA-2^*g*^ AmpC^*f*^	> 64	16	16
*Escherichia coli* GN688	CTX-M-15^*d*^ OXA-1^*g*^	TEM-1^*d*^ AmpC^*f*^	NDM-1^*e*^	16	8	8
*Klebsiella oxytoca* GN942	NDM-1^*e*^ OXA-9^*g*^	CTX-M-15^*d*^ OXY-2–8^*d*^	TEM-1^*d*^	8	32	8
*Klebsiella pneumoniae* 86500	SHV-11^*d*^ NDM-5 AmpH^*f*^	CTX-M-15^*d*^ OXA-1^*g*^	OXA-232^*g*^ TEM-1^*d*^	> 64	> 64	64
*Klebsiella pneumoniae* 110027	CTX-M-14^*d*^ OXA-48^*g*^ OXA-9^*g*^	NDM-1^*e*^ SHV-1^*d*^	CTX-M-15^*d*^ TEM-1^*d*^	> 64	> 64	16
*Klebsiella pneumoniae* 130392–2	DHA-1^*f*^ OXA-232^*g*^ OXA-9^*g*^	SHV-27^*d*^ CTX-M-15^*d*^ CMY-42^*f*^	NDM-1^*e*^ TEM-183^*d*^	16	64	16
*Klebsiella pneumoniae* 420322	SHV-1^*d*^ CTX-M-15^*d*^ AmpH^*f*^	OXA-181^*g*^ OXA-1^*g*^	NDM-5^*e*^ TEM-1^*d*^	> 64	> 64	64
*Klebsiella pneumoniae* GN529	TEM-1^*d*^ SHV-144^*d*^	NDM-1^*e*^ OXA-1^*g*^	CTX-M-15^*d*^ SHV-66^*d*^	8	> 64	8
*Klebsiella pneumoniae* GN629	SHV-11^*d*^ DHA-7^*f*^	CTX-M-15^*d*^ OXA-1^*g*^	NDM-1^*e*^	16	> 64	16
*Klebsiella pneumoniae* N11-0306	CTX-M-15^*d*^ OXA-1^*g*^	SHV-11^*d*^ DHA-7^*f*^	NDM-1^*e*^	16	> 64	16
*Klebsiella pneumoniae* N11-2218	NDM-1^*e*^ SHV-83^*d*^	CTX-M-15^*d*^ SHV-144^*d*^	CMY-6^*f*^ SHV-11^*d*^	8	> 64	8
*Morganella morganii* GN575	CTX-M-15^*d*^	NDM-1^*e*^	DHA-1^*f*^	8	> 64	8
*Providencia rettgeri* GN570	NDM-1^*e*^ CMY-6^*f*^	VEB-1a^*d*^ OXA-1^*g*^	TEM-1^*d*^	64	> 64	16
*Providencia stuartii* GN576	CMY-2^*f*^	NDM-1^*e*^		8	> 64	8
*Pseudomonas aeruginosa* 411090	NDM-1^*e*^ PDC-3^*f*^	VEB-9^*d*^ OXA-50^*g*^	OXA-10^*g*^	> 64	> 64	> 64
*Pseudomonas aeruginosa* H1010805	VIM-2^*e*^			> 64	> 64	> 64
*Pseudomonas aeruginosa* H1010812	IMP-7^*e*^	PDC-7^*f*^	OXA-485^*g*^	> 64	> 64	> 64

^*a*^ Sequencing data are available in the NCBI BioProject database under accession number PRJNA532924.

^*b*^ 2 µg/mL is the EUCAST susceptibility breakpoint concentration for MEM.

^*c*^ AVI was maintained at 4 µg/mL, except during the AVI/MEM combination.

^*d*^ Class A.

^*e*^ Class B.

^*f*^ Class C.

^*g*^ Class D.

^*h*^ AMA, aspergillomarasmine A; AVI, avibactam; MEM, meropenem.

^*i*^ All assays were conducted in duplicate.

## DISCUSSION

Although AMA has previously been shown to restore the activity of β-lactam antibiotics against MBL-expressing bacteria (14, 15), many bacterial strains produce multiple β-lactamases from different classes (7, 8). Therefore, tandem β-lactamase pGDP2 expression vectors were constructed to explore the inhibitory potency of the AMA/avibactam/β-lactam antibiotic combinations against 10 *E. coli* and 10 *K*. *pneumoniae* laboratory strains producing one MBL (NDM-1) and one SBL (KPC-2, CTX-M-15, CMY-2, OXA-23, or OXA-48) enzyme.

Generating plasmids with a promoter upstream of both β-lactamase genes resulted in similar antibiograms, regardless of the position of the genes in the constructs (Table S1). These results are consistent with previous studies, which demonstrated that adding a second promoter allowed equal expression of two genes in the same plasmid (28). The effectiveness of AMA and avibactam in combination with β-lactam antibiotics from three subclasses (penam, cephem, and carbapenem) could then be determined against bacterial strains containing these plasmids. Consistent with our previous study, AMA and avibactam demonstrated the most significant inhibitory potency when paired with a carbapenem antibiotic, such as meropenem or doripenem (Table 1), possibly due to β-lactam antibiotics having varying affinities for their targets, the penicillin-binding proteins (PBPs) (15). For example, carbapenems (e.g., meropenem and doripenem) are potent inhibitors of essential PBPs, such as PBP2, which is important for bacterial cell shape and elongation (29, 30). Comparatively, ampicillin’s bactericidal activity results from inhibiting several non-essential PBPs (30).

Consistent with the mode of action of AMA, the AMA/meropenem combination demonstrated no activity against SBL carbapenemases (e.g., KPC-2) in *E. coli* and *K. pneumoniae* laboratory strains (14). However, in the presence of AMA and avibactam, the activity of meropenem was restored against all laboratory strains, producing multiple β-lactamases (Tables 1 and 2). These results indicated that combining MBL and SBL inhibitors can restore β-lactam susceptibility against bacterial strains producing multiple β-lactamases. In addition, the inhibitory potency of the AMA/avibactam/meropenem combination did not appear to be pathogen-dependent for two representative Enterobacteriaceae laboratory strains.

The inhibitory potency of AMA, avibactam, and meropenem was also explored against 30 MDR clinical isolates. The AMA/meropenem and AMA/avibactam/meropenem combinations were most effective against Enterobacterales isolates producing SBLs from classes A and C and NDM-1 (Table 3). However, the AMA/meropenem combination was less effective against clinical isolates producing multiple class D OXA β-lactamases or NDM variants (e.g., NDM-5). Still, meropenem susceptibility generally increased upon adding avibactam to the combination. These results are consistent with previous studies demonstrating that AMA is a rapid and potent MBL inhibitor (14),whereas avibactam effectively inhibits class A, C, and some class D enzymes (27).

The AMA/meropenem and AMA/avibactam/meropenem combinations demonstrated little activity against *A. baumannii* and *P. aeruginosa* isolates regardless of their β-lactamase content (Table 3). This observed resistance could reflect the notable level of intrinsic and acquired antibiotic resistance mechanisms in *P. aeruginosa* (31). Furthermore, previous studies have shown that avibactam has little to no activity against carbapenem-hydrolyzing class D β-lactamases, such as OXA-23, OXA-24/40, OXA-48, OXA-58 and OXA-143 (27, 32), which are typically produced by *A. baumannii* (22, 23). Therefore, different SBL inhibitors may be required to alleviate *A. baumannii* and *P. aeruginosa* resistance. For example, durlobactam and taniborbactam have shown potent activity in clinical trials against MDR *Acinetobacter* spp. and carbapenem-resistant *P. aeruginosa*, respectively (27).

These results demonstrate that an AMA/avibactam/meropenem combination may have value in infections caused by Enterobacteriaceae producing class A, B, and C β-lactamase enzymes. As MDR isolates increasingly express many β-lactamases, higher-order combinations of antibiotics with β-lactamase inhibitors should be considered for further development to ensure adequate coverage of resistance mechanisms.

## MATERIALS AND METHODS

### Construction of pGDP2 vectors containing a promoter for each β-lactamase gene

The sequences of the β-lactamase genes were obtained from the Comprehensive Antibiotic Resistance Database (CARD) (33). The pGDP2 plasmids containing a single β-lactamase gene were constructed as described previously (15). These plasmids were used as the foundation for cloning the second β-lactamase gene into the pGDP2 vectors. The construction of the pGDP2:NDM-1/KPC-2 (Fig. S1) began with the polymerase chain reaction (PCR) amplification of *bla*_NDM-1_ from the pGDP2:NDM-1 plasmid using 5′–GCC AGC CTA GCC GGG AGA TCT–3′ as a forward primer and 5′–CCG TTG AGC ACC GCC GCC GCA GAA GGC CAT CCT GAC GGA TGG–3′ as the reverse primer. To facilitate gene expression in downstream experiments, the primers were designed to amplify the P_Lac_ promoter, the sequence of *bla*_NDM-1_, and the rrnB T2 terminator from the template plasmid. In addition, the forward and reverse primers were engineered to amplify approximately 20 nucleotides upstream and downstream, respectively, of the BglII recognition site of the pGDP2 vector. Gibson assembly was then employed to insert the purified *bla*_NDM-1_ DNA into a BglII digested pGDP2:KPC-2 plasmid, following the guidelines specified by the manufacturer (34).

Following XbaI digestion, the purified plasmid was transformed into chemically competent *E. coli* BW25113 or *K. pneumoniae* ATCC 33,495 cells to verify the second gene’s insertion. Transformation into *K. pneumoniae* cells was conducted using the freeze–thaw transformation procedure described in reference (35). The sequence of the plasmid was verified by Sanger sequencing. All other pGDP2 vectors containing two β-lactamase genes and two promoters were constructed as described above but using their respective β-lactamase genes.

### Cell-based antimicrobial assays

All cell-based assays were conducted in 96-well round base micro test plates (Sarstedt, Nümbrecht, Germany) based on the protocol described in references (15, 36). Most compounds employed in these assays were dissolved in water. However, AMA was diluted in water containing ≤5% (v/v) ammonium hydroxide (NH_4_OH), avibactam was dissolved in dimethyl sulfoxide (DMSO), and cefaclor was diluted in water containing 1 M hydrochloric acid (HCl). If water was used as a solvent, compounds were filter-sterilized before use.

For all the cell-based assays, a bacterial inoculum was prepared from the bacterial cells of interest using colonies picked from overnight plates whose optical density at 625 nm (OD_625_) was adjusted to 0.08–0.10. Once the optimal OD_625_ was reached, a 200-fold dilution of the inoculum in cation-adjusted Mueller Hinton II broth (CAMHB) was conducted before adding it to the micro test plate for a final assay volume of 100 µL.

For minimum inhibitory concentration (MIC) assays, 10 twofold dilutions of the β-lactam antibiotics or β-lactamase inhibitors (3.9–2,000 ng/mL, 0.5–256 µg/mL or 8–4,096 µg/mL) were added along the x-axis of the plate. Two columns were reserved for controls; one served as a growth control as it contained only bacterial inoculum, whereas the other was a sterility control containing only CAMHB. The MIC value was determined as the lowest concentration of β-lactam antibiotic or β-lactam inhibitor showing no bacterial growth.

Two-dimensional checkerboard assays were performed using AMA and meropenem or avibactam and meropenem. Briefly, twofold dilutions of AMA (2–32 µg/mL for *E. coli* BW25113 strains and 0.5–64 µg/mL for all other strains) or avibactam (1–16 µg/mL for *E. coli* BW25113 strains and 0.5–64 µg/mL for all other strains) were added along the x-axis. Twofold dilutions of meropenem (1–16 µg/mL for *E. coli* BW25113 strains and 0.5–64 µg/mL for all other strains) were then added along the y-axis of the plate. Four columns were reserved for controls; one contained twofold dilutions of AMA or avibactam, one possessed twofold dilutions of meropenem to verify MIC values, and the last two contained the bacterial inoculum and CAMHB to serve as growth and sterility controls. The efficacy of the combination was scored based on the minimum concentration of AMA or avibactam required to restore meropenem growth inhibition. According to the European Committee on Antimicrobial Susceptibility Testing (EUCAST), the susceptibility breakpoint concentration for meropenem is 2 µg/mL (26).

Potentiation assays were conducted with AMA, avibactam, and a β-lactam antibiotic. In brief, twofold dilutions of AMA (2–32 µg/mL for *E. coli* BW25113 strains and 0.5–64 µg/mL for all other strains) were added along the x-axis of a plate, whereas twofold dilutions of avibactam (1–16 µg/mL for *E. coli* BW25113 strains and 0.5–64 µg/mL for all other strains) were added along the y-axis of the plate. The β-lactam antibiotic was added to both plate axes at its EUCAST susceptibility breakpoint concentration (26). The susceptibility breakpoint concentrations for meropenem, doripenem, piperacillin, ampicillin, cefotaxime, cefepime, and cefaclor were 2, 1, 8, 8, 1, 1, and 1 µg/mL, respectively. Four columns of the plates were reserved for controls; three contained twofold dilutions of either AMA, avibactam, or the β-lactam antibiotic to verify MIC values, whereas one alternatively contained the bacterial inoculum and CAMHB to serve as growth and sterility controls. The efficacy of the combination was scored based on the concentration of AMA required to restore the activity of the different β-lactam antibiotics to their respective EUCAST susceptibility breakpoint concentration at 4 µg/mL of avibactam (26).

After a 16–20 h static incubation at 37°C, bioassay plates containing *E. coli* BW25113 were shaken for 5 min to resuspend the bacterial cells. However, bioassay plates containing all other strains were resuspended manually using a pipette to minimize the formation of aerosols. The bioassay plates were read spectrophotometrically at a wavelength of 600 nm using a BioTek Synergy H1 plate reader (BioTek, Winooski, VT). All cell-based assays were performed in duplicate.

### Genomic DNA extraction

LB medium was inoculated with the appropriate clinical strain. The inoculated medium was incubated at 37°C in a shaking incubator for 16–20 h. Cells were harvested by centrifugation (10,000×*g*, 3 min, room temperature) using a Fisher Scientific accuSpin Micro 17 microcentrifuge (Thermo Fisher Scientific, Waltham, MA). Cell pellets were resuspended in a combination of 180 µL of genomic digestion buffer [25 mM Tris-HCl, 2.5 mM EDTA, 1% (w/v) SDS, pH 8.0] and 20 µL of proteinase K (final concentration of 1–5 mg/mL). The resuspended cells were incubated at 55°C for 2 h with occasional mixing. The samples were supplemented with 20 µL of RNase A (final concentration of 1 mg/mL) and placed in a 37°C static incubator for 2 h. Following incubation, 200 µL of genomic lysis/binding buffer [30 mM Tris-HCl, 30 mM EDTA, 800 mM guanidine thiocyanate, 5% (v/v) Triton X-100, 5% (v/v) Tween 20, pH 8.0] was added, and the samples were mixed until a homogenous solution was obtained. A volume of 500 µL of phenol:chloroform:isoamyl alcohol (25:24:1) was then used to separate unwanted proteins and cellular debris from the genomic DNA. Following centrifugation (17,000×*g*, 5 min, room temperature), the genomic DNA, located in the top aqueous layer, was removed and placed in a new microcentrifuge tube. The phenol:chloroform:isoamyl alcohol extraction was repeated until no white precipitate existed between the organic and aqueous phases. The genomic DNA was then supplemented with a 0.1 vol of 3 M sodium acetate (pH 5.2) and one volume of cold 2-propanol. The tube was then gently inverted until a precipitate could be seen. Following centrifugation (12,000×*g*, 10 min, 4°C), the supernatant was removed, while ensuring that the precipitated DNA remained undisturbed. The pellet was washed with 1 mL of cold 70% (v/v) ethanol. The supernatant was removed after centrifugation (12,000×*g*, 10 min, 4°C). This ethanol wash was repeated twice. The pellet was then allowed to air dry before being resuspended in 50 µL of Tris-EDTA buffer. The purity of the genomic DNA was analyzed using a NanoDrop Spectrophotometer (Thermo Fisher Scientific) and a 1.0% (w/v) agarose gel. The genomes of the clinical strains were then sequenced by Illumina sequencing. Following sequencing, the genomes of the clinical strains were assembled, and resistance genes were identified by analyzing the genome assemblies using the Resistance Gene Identifier from the CARD, where only perfect and strict hits were retained (33).

## Data Availability

Sequencing data are available in the National Institutes of Health (NCBI) BioProject database under accession number PRJNA532924.

## SUPPLEMENTAL MATERIAL

Supplemental material aac.00272-24-s0001.docx Figure S1; Tables S1 to S4.

ASM does not own the copyrights to Supplemental Material that may be linked to, or accessed through, an article. The authors have granted ASM a non-exclusive, world-wide license to publish the Supplemental Material files. Please contact the corresponding author directly for reuse.

## References

[1] King DT, Sobhanifar S, Strynadka NCJ. 2016. One ring to rule them all: current trends in combating bacterial resistance to the β-lactams. Protein Sci 25:787–803. doi:10.1002/pro.2889 26813250 PMC4941212

[2] Blair JMA, Webber MA, Baylay AJ, Ogbolu DO, Piddock LJV. 2015. Molecular mechanisms of antibiotic resistance. Nat Rev Microbiol 13:42–51. doi:10.1038/nrmicro3380 25435309

[3] Shaw E, Rombauts A, Tubau F, Padullés A, Càmara J, Lozano T, Cobo-Sacristán S, Sabe N, Grau I, Rigo-Bonnin R, Dominguez MA, Carratalà J. 2018. Clinical outcomes after combination treatment with ceftazidime/avibactam and aztreonam for NDM-1/OXA-48/CTX-M-15-producing Klebsiella pneumoniae infection. J Antimicrob Chemother 73:1104–1106. doi:10.1093/jac/dkx496 29272413

[4] Gonzales PR, Pesesky MW, Bouley R, Ballard A, Biddy BA, Suckow MA, Wolter WR, Schroeder VA, Burnham C-A, Mobashery S, Chang M, Dantas G. 2015. Synergistic, collaterally sensitive β-lactam combinations suppress resistance in MRSA. Nat Chem Biol 11:855–861. doi:10.1038/nchembio.1911 26368589 PMC4618095

[5] Nikaido H. 2009. Multidrug resistance in bacteria. Annu Rev Biochem 78:119–146. doi:10.1146/annurev.biochem.78.082907.145923 19231985 PMC2839888

[6] Bebrone C. 2007. Metallo-beta-lactamases (classification, activity, genetic organization, structure, zinc coordination) and their superfamily. Biochem Pharmacol 74:1686–1701. doi:10.1016/j.bcp.2007.05.021 17597585

[7] Rawat D, Nair D. 2010. Extended-spectrum β-lactamases in Gram negative bacteria. J Glob Infect Dis 2:263–274. doi:10.4103/0974-777X.68531 20927289 PMC2946684

[8] Mhlongo N, Essack S, Govinden U. 2015. NDM-1, novel TEM-205, novel TEM-213 and other extended-spectrum β-lactamasesco-expressed in isolates from cystic fibrosis patients from South Africa. South Afr J Infect Dis 30:103–107. doi:10.1080/23120053.2015.1074441

[9] Tamma PD, Cosgrove SE, Maragakis LL. 2012. Combination therapy for treatment of infections with gram-negative bacteria. Clin Microbiol Rev 25:450–470. doi:10.1128/CMR.05041-11 22763634 PMC3416487

[10] Palzkill T. 2013. Metallo-β-lactamase structure and function. Ann N Y Acad Sci 1277:91–104. doi:10.1111/j.1749-6632.2012.06796.x 23163348 PMC3970115

[11] Bush K, Bradford PA. 2019. Interplay between β-lactamases and new β-lactamase inhibitors. Nat Rev Microbiol 17:295–306. doi:10.1038/s41579-019-0159-8 30837684

[12] Rotondo CM, Wright GD. 2017. Inhibitors of metallo-β-lactamases. Curr Opin Microbiol 39:96–105. doi:10.1016/j.mib.2017.10.026 29154026

[13] Tehrani K, Martin NI. 2018. β-lactam/β-lactamase inhibitor combinations: an update. Med Chem Commun 9:1439–1456. doi:10.1039/C8MD00342D PMC6151480 30288219

[14] King AM, Reid-Yu SA, Wang W, King DT, De Pascale G, Strynadka NC, Walsh TR, Coombes BK, Wright GD. 2014. Aspergillomarasmine A overcomes metallo-β-lactamase antibiotic resistance. Nature 510:503–506. doi:10.1038/nature13445 24965651 PMC4981499

[15] Rotondo CM, Sychantha D, Koteva K, Wright GD. 2020. Suppression of β-lactam resistance by aspergillomarasmine A is influenced by both the metallo-β-lactamase target and the antibiotic partner. Antimicrob Agents Chemother 64:e01386-19. doi:10.1128/AAC.01386-19 31932375 PMC7179287

[16] Ehmann DE, Jahic H, Ross PL, Gu R-F, Hu J, Durand-Réville TF, Lahiri S, Thresher J, Livchak S, Gao N, Palmer T, Walkup GK, Fisher SL. 2013. Kinetics of avibactam inhibition against class A, C, and D β-lactamases. J Biol Chem 288:27960–27971. doi:10.1074/jbc.M113.485979 23913691 PMC3784710

[17] Cox G, Sieron A, King AM, De Pascale G, Pawlowski AC, Koteva K, Wright GD. 2017. A common platform for antibiotic dereplication and adjuvant discovery. Cell Chem Biol 24:98–109. doi:10.1016/j.chembiol.2016.11.011 28017602

[18] Mehta SC, Rice K, Palzkill T. 2015. Natural variants of the KPC-2 carbapenemase have evolved increased catalytic efficiency for ceftazidime hydrolysis at the cost of enzyme stability. PLoS Pathog 11:e1004949. doi:10.1371/journal.ppat.1004949 26030609 PMC4452179

[19] Poirel L, Nordmann P, Ducroz S, Boulouis HJ, Arné P, Millemann Y. 2013. Extended-spectrum β-lactamase CTX-M-15-producing Klebsiella pneumoniae of sequence type ST274 in companion animals. Antimicrob Agents Chemother 57:2372–2375. doi:10.1128/AAC.02622-12 23422912 PMC3632922

[20] Dortet L, Poirel L, Nordmann P. 2014. Worldwide dissemination of the NDM-type carbapenemases in Gram-negative bacteria. Biomed Res Int 2014:249856. doi:10.1155/2014/249856 24790993 PMC3984790

[21] Guo YF, Zhang WH, Ren SQ, Yang L, Lü DH, Zeng ZL, Liu YH, Jiang HX. 2014. IncA/C plasmid-mediated spread of CMY-2 in multidrug-resistant Escherichia coli from food animals in China. PLoS One 9:e96738. doi:10.1371/journal.pone.0096738 24816748 PMC4016023

[22] Poirel L, Potron A, Nordmann P. 2012. OXA-48-like carbapenemases: the phantom menace. J Antimicrob Chemother 67:1597–1606. doi:10.1093/jac/dks121 22499996

[23] Antunes NT, Lamoureaux TL, Toth M, Stewart NK, Frase H, Vakulenko SB. 2014. Class D β-lactamases: are they all carbapenemases? Antimicrob Agents Chemother 58:2119–2125. doi:10.1128/AAC.02522-13 24468778 PMC4023754

[24] Singkham-In U, Muhummudaree N, Chatsuwan T. 2021. In vitro synergism of azithromycin combination with antibiotics against OXA-48-producing Klebsiella pneumoniae Clinical Isolates. Antibiotics (Basel) 10:1551. doi:10.3390/antibiotics10121551 34943763 PMC8698995

[25] Jacoby GA. 2009. AmpC β-lactamases. Clin Microbiol Rev 22:161–182. doi:10.1128/CMR.00036-08 19136439 PMC2620637

[26] The European Committee on Antimicrobial Susceptibility Testing. 2024. Version 14.0. Breakpoint tables for interpretation of MICs and zone diameters. Available from: https://www.eucast.org/clinical_breakpoints/. Retrieved Feb 2024.

[27] Lence E, González‐Bello C. 2021. Bicyclic boronate β‐lactamase inhibitors: the present hope against deadly bacterial pathogens. Advanced Therapeutics 4:2000246. doi:10.1002/adtp.202000246

[28] Kim K-J, Kim H-E, Lee K-H, Han W, Yi M-J, Jeong J, Oh B-H. 2004. Two-promoter vector is highly efficient for overproduction of protein complexes. Protein Sci 13:1698–1703. doi:10.1110/ps.04644504 15133160 PMC2279994

[29] Legaree BA, Daniels K, Weadge JT, Cockburn D, Clarke AJ. 2007. Function of penicillin-binding protein 2 in viability and morphology of Pseudomonas aeruginosa. J Antimicrob Chemother 59:411–424. doi:10.1093/jac/dkl536 17289762

[30] Kocaoglu O, Carlson EE. 2015. Profiling of β-lactam selectivity for penicillin-binding proteins in Escherichia coli strain DC2. Antimicrob Agents Chemother 59:2785–2790. doi:10.1128/AAC.04552-14 25733506 PMC4394777

[31] Pang Z, Raudonis R, Glick BR, Lin TJ, Cheng Z. 2019. Antibiotic resistance in Pseudomonas aeruginosa: mechanisms and alternative therapeutic strategies. Biotechnol Adv 37:177–192. doi:10.1016/j.biotechadv.2018.11.013 30500353

[32] Lahiri SD, Mangani S, Jahić H, Benvenuti M, Durand-Reville TF, De Luca F, Ehmann DE, Rossolini GM, Alm RA, Docquier J-D. 2015. Molecular basis of selective inhibition and slow reversibility of avibactam against class D carbapenemases: a structure-guided study of OXA-24 and OXA-48. ACS Chem Biol 10:591–600. doi:10.1021/cb500703p 25406838

[33] Alcock BP, Raphenya AR, Lau TTY, Tsang KK, Bouchard M, Edalatmand A, Huynh W, Nguyen A-LV, Cheng AA, Liu S, et al.. 2020. CARD 2020: antibiotic resistome surveillance with the comprehensive antibiotic resistance database. Nucleic Acids Res 48:D517–D525. doi:10.1093/nar/gkz935 31665441 PMC7145624

[34] New England BioLabs. 2012. Gibson assembly protocol (E5510). Available from: https://international.neb.com/protocols/2012/12/11/gibson-assembly-protocol-e5510. Retrieved Feb 2022.

[35] Merrick MJ, Gibbins JR, Postgate JR. 1987. A rapid and efficient method for plasmid transformation of Klebsiella pneumoniae and Escherichia coli. Microbiology 133:2053–2057. doi:10.1099/00221287-133-8-2053 3327913

[36] Clinical and Laboratory Standards Institute. 2012. Methods for dilution antimicrobial susceptibility tests for bacteria that grow aerobically. 9th ed. CLSI, Wayne, PA.

