# Supplementary material for: Efficacy of aspergillomarasmine A/meropenem combinations with and without avibactam against bacterial strains producing multiple β-lactamases
Source: Antimicrob Agents Chemother. 2024 Aug 12;68(9):e00272-24. doi: 10.1128/aac.00272-24 (PMC11373191; doi:10.1128/aac.00272-24)
Supplement: Supplemental material — Figure S1; Tables S1 to S4. [file aac.00272-24-s0001.docx]

**SUPPLEMENTAL MATERIAL**

Efficacy of AMA/meropenem combinations with and without avibactam against bacterial strains producing multiple β-lactamases

Caitlyn M. Rotondo,^a,b,c^ Gerard D. Wright^a,b,c,*^

^a^ David Braley Centre for Antibiotic Discovery, McMaster University, Hamilton, Ontario, Canada

^b^ M.G. DeGroote Institute for Infectious Disease Research, McMaster University, Hamilton, Ontario, Canada

^c^ Department of Biochemistry and Biomedical Sciences, McMaster University, Hamilton, Ontario, Canada

^*^ Address correspondence to wrightge@mcmaster.ca

**SUPPLEMENTAL FIGURE 1** Plasmid map for the NDM-1/KPC-2 construct under individual promoter control. The positions of the β-lactamase genes in the plasmid are depicted in red and blue. The restriction sites used to clone the genes within the plasmid are also portrayed. Plasmid map was created and annotated using SnapGene Viewer (Dotmatics, Boston, MA).

**SUPPLEMENTAL TABLE 1** Minimum inhibitory concentration (MIC) values of *E. coli* BW25113 strains producing one MBL and one SBL when tested against various β-lactam antibiotics.^a^ Genes from class A are shown in blue, while genes from class B, class C, and class D are depicted in black, pink, and green. All assays were conducted in duplicate.

| **β-Lactamase ^b^** | | **MIC values (µg/mL)** | | | | | | | | |
| --- | --- | --- | --- | --- | --- | --- | --- | --- | --- | --- |
| **Gene 1** | **Gene 2** | **ATM** | **AVI** | **MEM** | **DOR** | **PIP** | **AMP** | **CTX** | **FEP** | **CEC** |
| NDM-1 | KPC-2 | 128 | 16 | 64 | 32 | 2048 | > 4096 | 256 | 64 | 512 |
| NDM-1 | CTX-M-15 | 128 | 16 | 64 | 32 | 4096 | > 4096 | 4096 | 1024–2048 | 1024 |
| NDM-1 | CMY-2 | 32 | 8 | 64 | 32 | 512 | > 4096 | 256 | 32 | 512–1024 |
| NDM-1 | OXA-23 | 0.25 | 16–32 | 128 | 64 | 1024 | > 4096 | 256–512 | 128 | 512 |
| NDM-1 | OXA-48 | 0.13 | 8 | 64 | 32 | 512 | > 4096 | 256 | 32 | 512 |

Abbreviations: ATM aztreonam, AVI avibactam, MEM meropenem, DOR doripenem, PIP piperacillin, AMP ampicillin, CTX cefotaxime, FEP cefepime, CEC cefaclor.

^a^ Both β-lactamase genes were cloned into pGDP2 with individual promoters.

^b^ As similar results were obtained regardless of the position of the β-lactamase genes, the data for the constructs with NDM-1 at position 2 were removed from the table.

**SUPPLEMENTAL TABLE 2** Minimum inhibitory concentration (MIC) values of *E. coli* BW25113 strains producing a single β-lactamase gene when tested against various β-lactam antibiotics.^a^ Genes from class A are shown in blue, while genes from class B, class C, and class D are depicted in black, pink, and green. *E. coli* BW25113 transformed with empty pGDP2 plasmid was used as a control. All assays were conducted in duplicate.

| **β-**  **Lactamase** |  | **MIC values (µg/mL)** | | | | | | | |
| --- | --- | --- | --- | --- | --- | --- | --- | --- | --- |
|  | **ATM** | **AVI** | **MEM** | **DOR** | **PIP** | **AMP** | **CTX** | **FEP** | **CEC** |
| None | 0.13 | 8–16 | 0.06 | 0.03 | 2–4 | 8 | 0.06 | 0.06–0.013 | 4 |
| NDM-1 | 0.13 | 16 | 32–64 | 32 | 256 | > 4096 | 256 | 32 | 256 |
| KPC-2 | 128 | 16–32 | 8 | 4 | 512 | 4096 | 128–256 | 8 | 1024 |
| CTX-M-15 | 128 | 16 | 0.06 | 0.06 | 1024 | > 4096 | 512 | 64 | 512 |
| CMY-2 | 32 | 16–32 | 0.06 | 0.06 | 32–64 | 512 | 2 | 0.25–0.50 | 512 |
| OXA-23 | 0.25 | 16 | 0.25 | 0.06 | 128 | 1024 | 0.06 | 0.13–0.25 | 32 |
| OXA-48 | 0.13 | 16 | 1 | 0.06 | 64–128 | 4096 | 512 | 0.13 | 64 |

Abbreviations: ATM aztreonam, AVI avibactam, MEM meropenem, DOR doripenem, PIP piperacillin, AMP ampicillin, CTX cefotaxime, FEP cefepime, CEC cefaclor

^a^ All the β-lactamase genes were cloned into the pGDP2 vector.

**SUPPLEMENTAL TABLE 3** Minimum inhibitory concentration (MIC) values of *K. pneumoniae* ATCC 33495 producing a single β-lactamase gene when tested against various β-lactam antibiotics.^a^ Genes from class A are shown in blue, while genes from class B, and D are depicted in black, and green. *K. pneumoniae* ATCC 33495 transformed with empty pGDP2 plasmid was used as a control. All assays were conducted in duplicate.

| **β-**  **Lactamase** |  | **MIC values (µg/mL)** | | | | | | | |
| --- | --- | --- | --- | --- | --- | --- | --- | --- | --- |
|  | **ATM** | **AVI** | **MEM** | **DOR** | **PIP** | **AMP** | **CTX** | **FEP** | **CEC** |
| None | 0.06 | 32 | 0.06 | 0.06 | 8 | 64 | 0.06 | 0.06 | 0.50–1 |
| NDM-1 | 0.06 | 512–1024 | 128 | 256–512 | 512 | > 4096 | 256 | 32 | 1024 |
| CTX-M-15 | 64–128 | 1024 | 0.06 | 0.13 | 2048 | > 4096 | 4096 | 32 | > 4096 |
| OXA-23 | 0.06–0.13 | 256 | 0.25 | 256 | 256 | 2048 | 0.06–0.13 | 0.13 | 128 |
| OXA-48 | 0.06 | 256 | 0.25 | 64 | 128–256 | 1024–2048 | 0.50 | 0.06–0.13 | 128 |

Abbreviations: ATM aztreonam, AVI avibactam, MEM meropenem, DOR doripenem, PIP piperacillin, AMP ampicillin, CTX cefotaxime, FEP cefepime, CEC cefaclor.

^a^ All the β-lactamase genes were cloned into the pGDP2 vector.

**SUPPLEMENTAL TABLE 4** Minimum inhibitory concentration (MIC) values of clinical strains. All assays were conducted in duplicate.

| **Strain** | **MIC values (µg/mL)** | |
| --- | --- | --- |
|  | **MEM** | **AVI** |
| *Acinetobacter baumannii* B1NG08a | 256 | > 4096 |
| *Citrobacter freundii* GN978 | 32 | 64 |
| *Enterobacter cloacae* 36749 | 8–16 | 16 |
| *Enterobacter cloacae* 47219 | 64 | 32 |
| *Enterobacter cloacae* 86502 | 128 | 32 |
| *Enterobacter cloacae* 397260 | 16 | 64–128 |
| *Enterobacter cloacae* GN574 | 128–256 | 128 |
| *Enterobacter cloacae* GN579 | 32 | 32 |
| *Enterobacter cloacae* GN687 | 128 | 64 |
| *Escherichia coli* 130392-1 | 128 | 32–64 |
| *Escherichia coli* 376762 | 128 | 128 |
| *Escherichia coli* 376948 | 256 | 16 |
| *Escherichia coli* 387039 | 256 | 64 |
| *Escherichia coli* GN610 | 512 | 16 |
| *Escherichia coli* GN688 | 128 | 16 |
| *Klebsiella oxytoca* GN942 | 128 | 64 |
| *Klebsiella pneumoniae* 86500 | 512 | 128–256 |
| *Klebsiella pneumoniae* 110027 | 1024 | 256–512 |
| *Klebsiella pneumoniae* 130392-2 | 128 | 256–512 |
| *Klebsiella pneumoniae* 420322 | 512 | > 4096 |
| *Klebsiella pneumoniae* GN529 | 128–256 | 256–512 |
| *Klebsiella pneumoniae* GN629 | 64–128 | 512 |
| *Klebsiella pneumoniae* N11-0306 | 64–128 | 256 |
| *Klebsiella pneumoniae* N11-2218 | 128 | 256–512 |
| *Morganella morganii* GN575 | 16–32 | > 4096 |
| *Providencia rettgeri* GN570 | 128 | 1024 |
| *Providencia stuartii* GN576 | 64 | > 4096 |
| *Pseudomonas aeruginosa* 411090 | 2048 | > 4096 |
| *Pseudomonas aeruginosa* H1010805 | 256 | 4096 |
| *Pseudomonas aeruginosa* H1010812 | 1024–2048 | > 4096 |

Abbreviations: MEM meropenem, AVI avibactam.
